# Pig Manure Management: A Methodology for Environmentally Friendly Decision-Making

**DOI:** 10.3390/ani12060747

**Published:** 2022-03-16

**Authors:** Andrey Izmaylov, Aleksandr Briukhanov, Ekaterina Shalavina, Eduard Vasilev

**Affiliations:** Federal Scientific Agroengineering Center VIM, Branch in Saint Petersburg, 196625 Saint Petersburg, Russia; vim@vim.ru (A.I.); nii@sznii.ru (A.B.); sznii6@yandex.ru (E.V.)

**Keywords:** pig slurry, nutrients, processing technology, end-product, organic fertiliser

## Abstract

**Simple Summary:**

Intensification of pig farming increases the profitability of farms but also leads to greater local environmental pressure from manure nutrient loss. There are various technologies for pig manure processing. Each technological solution provides specific nutrient content in the end-products (organic fertilisers, effluents, biogas, etc.). The methodology was developed for choosing proper technological solutions for pig farms with due account for the whole variety of combinations of production and natural and climatic conditions. It includes an accurate accounting for nitrogen and phosphorus flows in the manure processing and utilisation chain and considers the limiting factors of the farm and the end-product customer requirements. The methodology was applied to a large-scale pig farm in the Leningrad Region. The best suitable processing technology was identified demonstrating the total amount of nitrogen and phosphorus in the end-products of 278.94 t per year. The developed methodology can serve as an effective tool to reduce manure nutrient loss and mitigate the adverse effect on the rural environment.

**Abstract:**

The current trend towards larger pig farms increases their profitability but might harm animal welfare and the environment. More efficient pig manure management is a way to address this challenge. Available manure handling and utilisation systems may feature from 8 to 50% nutrient loss to the environment. Therefore, the proper choice of technological solutions is of high priority. In regard, the study developed a methodology including calculation, analysis and modelling techniques for the accurate accounting of manure amount, its fractions and their nitrogen and phosphorus content for different processing technologies with due regard to the limiting factors of the farm and the end-product consumer requirements. The methodology was applied to justify the best suitable processing technology for a large-scale pig farm in the Leningrad Region with 17,800 heads of pigs and 54,750 t of pig manure per year. The selected technology included manure separation into fractions, aeration and secondary sedimentation of the liquid fraction and passive composting of the solid fraction. It demonstrated the total amount of nitrogen (N) and phosphorus (P) in the end-products of 278.94 t per year, with the 26% total nutrients loss on all technological stages, and specific capital and operating costs of USD 55.5 per ton of manure produced. The methodology was tested by comparing the calculated data and the data from previous surveys of 15 pig farms in Russia. The differences between the values were found from 0.9 to 12.5% in mass; 2.8 to 13.9% in N content and 1.7 to 9.9% in P content. The developed methodology supports planning the production of the manure-based end-products in a given amount and with target nutrient content, depending on different processing technological solutions, achieving both economic and environmental goals.

## 1. Introduction

Intensification of pig husbandry raises the farms’ profitability, but it also entails a greater load on the local environment and may adversely affect animal welfare. The analysis revealed that nearly all intensive pig farms in Russia, especially large-scale pig rearing complexes, require their operation to be optimally improved in terms of economic profitability and minimal impact on the environment [1]. Manure handling systems require special attention in this respect [2].

Manure nutrients (total nitrogen and total phosphorus) are considered the main source of environmental pollution, on the one hand, but they maintain soil fertility, on the other. However, currently, 47% of pig farms in the country have neither enough land to apply all organic fertilisers produced nor any arrangements with crop growing farms on fertiliser export [3,4]. In this context, decisions must be taken at the district or regional levels for the redistribution of organic fertilisers between farms. Most efficient manure processing is required to achieve the minimal nutrient loss and the cost-effective transportation. This ambition is a cornerstone of modern technological schemes for multi-stage pig manure processing. To meet it, environmentally and economically sound technologies need to be chosen and adapted to particular farm conditions for consequent managerial decision-making.

Researchers in different countries offer their solutions to the problem of how to make intensive pig farming more environmentally sound. These solutions include, among others, the manure nutrients management system based on the mass balance method [5,6,7,8], energy-saving approaches [9,10] and intelligent technologies that would provide the automated process monitoring, data analysis and technological and managerial decision-making [11,12,13]. The life cycle assessment is a tool widely used in assessing the environmental performance of integrated systems, such as large-scale pig farms [14,15,16].

The accurate nutrient accounting requires calculation methods of the quantity and quality of pig manure and resulting organic fertilisers and other by-products [17,18,19,20].

A technological solution for pig manure processing is selected based on the technical and land resources of the farm and the end-product requirements of customers. However, any technological solution is expected to apply all tools to minimize emissions in the production process [21,22,23]. Pig slurry acidification is one of the practices to reduce hazardous emissions [24].

One way to optimise the nutrient flow is to separate the pig manure into solid and liquid fractions [25,26,27] and treat them further individually [28,29] to obtain, among others, high-quality organic fertilisers. Researchers note that the techniques of primary separation of pig manure into fractions strongly affect the plant uptake of nutrients supplied with organic fertilisers [30]. Special research attention is paid to the methods of producing organic fertilisers and biogas [31,32].

It is important to consider the nutrients produced both at the farm level and in larger agroecosystems at a district or region level [33,34,35]. In this respect, the sampling technique also plays an important role in the nutrients inventory [36]. The organic fertilisers produced from the pig slurry should be applied to the soil with the least loss to ensure soil fertility improvement and the harvest of target yields [37,38].

However, if a large-scale pig farm has no agricultural land, the use of pig manure as a liquid organic fertiliser is uneconomical. It is worth considering other end-product options. In a concentrated form, they might be more cost-effective organic fertilisers. While the nutrient-poor cleaned water can be reused at the farm.

The scientific hypothesis of the work is that the environmental sustainability of a pig farm is achieved, among others, by creating a balanced system of the effective transfer (flow) of nutrients (nitrogen and phosphorus) from the pig manure into the end-products. An effective tool in this respect is to choose the technological solutions that consider the characteristics of both the source material and the target products (solid and liquid organic fertilisers, biogas, cleaned water, etc.).

The study objective was to assess the environmental and economic performance of a large-scale intensive pig farm located in the Baltic Sea catchment area. For this purpose, a special tool (methodology) was designed to support the choice of such a technological solution to manure processing that would be best suited in terms of available resources, the end-product requirements and environmental effect.

The methodology will include the calculation techniques for the quantity and quality of the fresh manure and the end-products (in terms of the customer needs), the computational analysis of various technological solutions to converting manure into the end-products, the modelling of mass and nutrient distribution and the estimation of relevant costs.

## 2. Materials and Methods

Following the study aim, intensive pig farming in the Russian part of the Baltic Sea Region was considered using a systemic approach.

### 2.1. Large-Scale Pig Farm as an Argoecosystem

A present-day large-scale pig farm, as a rule, has several production sites and may cover a vast area since its numerous production facilities and fields are located at a substantial distance from each other.

Therefore, such a farm may be regarded as a separate integrated agroecosystem. Its sustainability is of great importance for both the farm itself and the environment.

**Figure 1:**
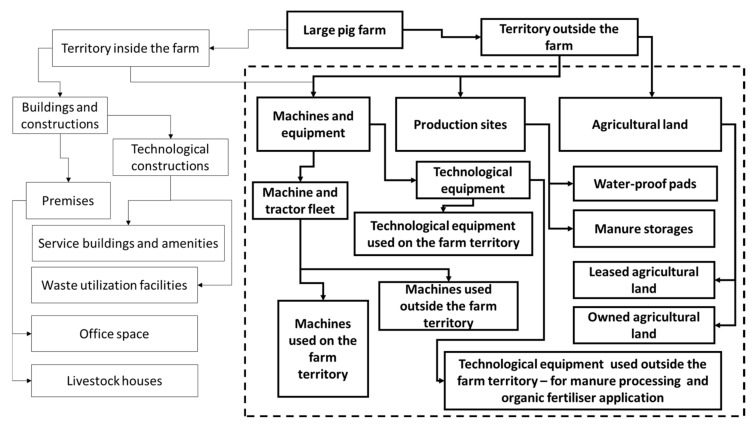
The structure of a large pig farm.

Such a farm has the sources of point pollution (buildings, constructions, machinery and equipment), non-point pollution (fields with applied organic fertilisers) and combined pollution (production sites, where the pig manure is processed into organic fertilisers) (Figure 1).

Long-term experience of research associated with technological solutions for pig farms allows dividing them into three types.

1. A closed-type large-scale pig farm is a self-sufficient “mono-system” where production and processing take place right on the farm without involving external players. Its characteristic features are sufficient capacity of pig manure processing sites and storages; enough agricultural land within a cost-effective transportation distance to apply all organic fertilisers produced; the owned feed mill and meat-processing facility; and cultivation of crops for the own use for feed and sale, and direct selling of pork and by-products.
2. A combined-type large-scale pig farm is a “mono-system” with minimal involvement of external players. In contrast to the closed-type farm, it does not have a feed mill and purchases the feeds. Available agricultural land is not enough to apply all organic fertilisers produced. Therefore, such a farm rents some land for fertiliser application that sometimes leads to uneconomic transportation distances.
3. An open-type pig farm is a “mono-system” involving many external players. It differs from the combined-type farm in that the pig manure processing and organic fertiliser field application are passed to third-party organisations.

A comprehensive analysis of available technological solutions to pig manure handling allows dividing them by the obtained end-products into three groups: single-product technological solutions, two-product technological solutions and multi-product technological solutions.

The output of single-product technological solutions is the solid or liquid organic fertilisers, with the target being the maximum saving of nutrients (minimum nutrients loss).

The output of two-product technological solutions is an organic fertiliser (solid or liquid) and water (effluent) that can be used for additional fertilisation of crops or process needs. After additional biological treatment, it can be discharged into the open water bodies if its chemical composition complies with established standards. The target of such technological solutions is to concentrate nutrients in the organic fertiliser to reach the required quality of the cleaned water.

**Figure 2:**
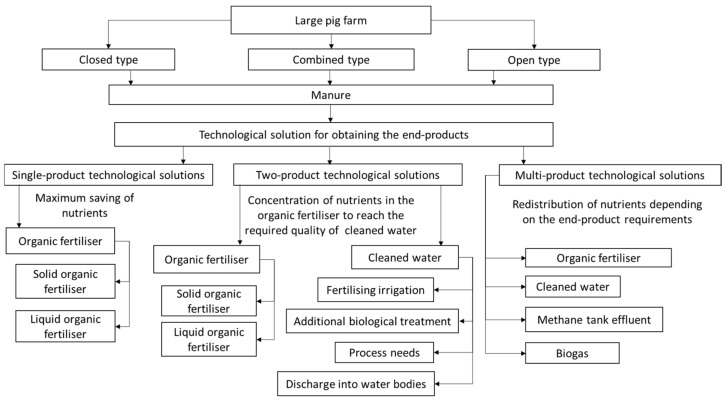
Methodical approach to obtaining quantity and quality of end-products depending on applied technological solutions.

The output of multi-product technological solutions is organic fertilisers (solid or liquid), cleaned water and other end-products: biogas, electric energy, methane tank effluent and others. The target of such technological solutions is the redistribution of nutrients depending on the end-product requirements (Figure 2).

Characteristics of raw manure to be processed and the end-products (mass, moisture content, total nitrogen and phosphorus content) are considered separately for each technology.

### 2.2. Methods, Procedures and Models

The study integrated several previously worked out methods, including those of other researchers, in the methodology under development. The methodology is understood as a body of methods, approaches and principles employed to reach the research objectives.

The study of two-product and multi-product technological solutions included the survey of design documents, scientific reports and publications for 1970–1990. Those years, all large pig rearing complexes under construction in the Russian Federation and Eastern Europe widely introduced the technologies for multi-stage processing of the liquid fraction of pig manure and fermentation of the solid fraction. The obtained data were used to calculate the basic coefficients in the models describing technological processes. Due to the recent changes in animal diets and manure removal solutions, additional laboratory and farm-scale studies were performed to update the basic coefficients.

Mathematical models were obtained by the method of least squares of the regression equation. The resulting models and coefficients are statistically significant. The statistical significance of the models was tested by Fisher’s criterion and determination coefficient. The statistical significance of the coefficients was checked by Student’s *t*-test. The coefficients values reflect the influence degree of factors on the dependent variable.

The study applied theoretical methods of functional and structural analysis, decomposition of technological processes, expert evaluation method, mathematical methods of optimisation, theoretical modelling methods and laboratory experiments on pig manure from selected pig farms.

The experimental data were analysed in Microsoft Excel 2016 and Statgraphics Centurion 16.1.11 software packages.

**Figure 3:**
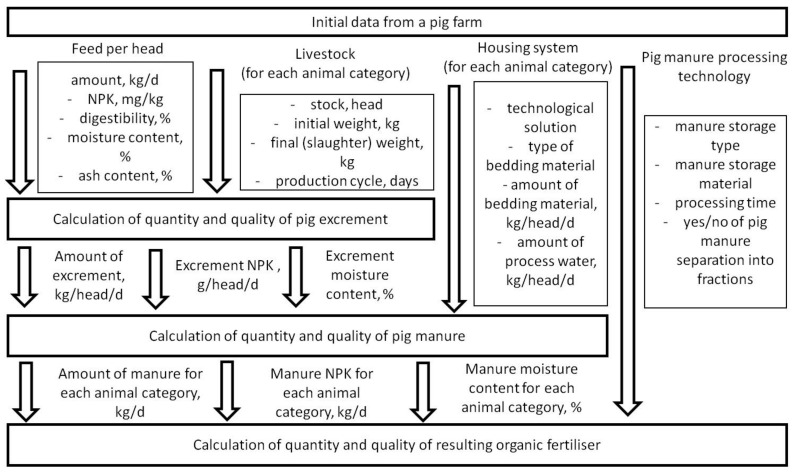
Sequence of calculating the quantity and quality of the pig manure as an initial material for obtaining the end-products—organic fertilisers.

Figure 3 shows the sequence of calculating the quantity and quality of both pig manure to be processed and the end-products.

#### 2.2.1. Methods for Calculating the Quantity and Quality of the Initial Material for the End Products Production

The calculations apply the mass balance method [5,6,7,8]. The amount of animal excrement is determined from the pigs’ diets and feed compositions, planned weight gains, nutrient content in the animal body, production cycles, etc. (Figure 3). The results are further used to calculate the ex-house manure with due account for the composition and amount of the applied bedding material and the process water that gets into the excrement. The source data required for calculations are obtained from the survey of pilot pig farms. The quantity and quality of pig manure depend on the pig housing system and technological solutions for manure removal from pig houses.

#### 2.2.2. Methods for Calculating the Quantity and Quality of the End-Products

The data received on the pig manure amount and its nutrient (nitrogen and phosphorus) content is used to calculate the quantity and quality of resulting organic fertilisers with due account for specific features of processing technologies in place in the Russian part of the Baltic Sea Region. Reasonable nutrient loss factors are used for each stage of manure and fertiliser handling—transportation of manure from livestock houses to either processing, temporary accumulation place, or both, transportation of end-products and organic fertiliser, use of end-products, and application of organic fertilisers, with the specific values of the factors being found in [1,5,7,8,18,22]. The mass of total nitrogen and phosphorus in the end products is calculated as the difference between the initial content and the sum of losses.

The procedure includes the models based on the principle of efficient use of nitrogen and phosphorus in the farm operation chain [39,40].

Nitrogen use efficiency (*NUE*) is calculated as

(1) $$NUE=\frac{\sum N_{output}}{\sum N_{input}}\times 100\%$$

Phosphorus use efficiency (PUE) is calculated in a similar way.

The procedure also applies the mathematical models for calculating the concentration of nutrient content in organic fertilisers to achieve the target quality of cleaned water. They contain the coefficients characterising the operating modes and process parameters of equipment and calculated coefficients characterising required quantity and quality of each type of end-products [1,3,41,42,43,44].

#### 2.2.3. The Designing Method of Pig Manure Processing Technologies to Obtain the Target End-Products with the Required Quality

The method involves the use of mathematical models, which describe the most common and emerging technological solutions for pig manure processing: long-term storing (maturing), passive and active composting, fermentation in drum and chamber fermenters and multi-stage processing with biological treatment [45,46,47,48,49,50].

The calculations associated with well-researched technological solutions and processes used the known formulas and dependencies [1,41,44,45,47].

For the under-studied technological solutions, the experimental studies were performed to obtain the coefficients required in the calculations.

##### The Multi-Stage Processing of the Liquid Fraction of Pig Manure

The subject of a separate laboratory-scale experiment was three-stage processing of the liquid fraction of pig manure. Stage 1 was sedimentation in a vertical settling tank. Stage 2 was aeration of the supernatant (clear water) followed by sedimentation in a settling tank. Stage 3 was the long-term storing (maturing) of the mixture of sludge from Stage 1 and excess activated sludge from stage 2 [51].

The results of the experiment were used to establish dependencies between the amount of cleaned water, its total nitrogen and total phosphorus content and the duration of technological processes (Formulas (2)–(5)):

(2) $$X1_{2}=M1-\frac{M1}{100}\times \left(102.5-39.5\times \sqrt{t_{22}}\right)$$

where

$X1_{2}$—mass of cleaned water, t
M1—mass of cleaned water and activated sludge after aeration, t
$t_{22}$—secondary sedimentation time, h

(3) $$M1=\left(Q-X1_{1}+A\times 10^{-3}+t_{21}\times 0.2\times 10^{-3}\right)-L1$$

where

M1—mass of cleaned water and activated sludge after aeration, t
Q—mass of the liquid fraction of pig manure, t d^−1^
$X1_{1}$—mass of sludge depending upon the primary sedimentation time, kg
L1—loss of mass of clear water (supernatant) and activated sludge during aeration, t
A—mass of activated sludge initially added to the aeration tank, kg
$t_{21}$—aeration time, d

(4) $$M2=\frac{N\times Q\times 10^{-6}+m2-X2_{1}}{100}\times \sqrt{-1387.13+1828.5\times \sqrt{t_{21}}}$$

where

Q—mass of the liquid fraction of pig manure, t d^−1^
$t_{21}$—aeration time, d
M2—mass of total nitrogen in the cleaned water and activated sludge after aeration, t
N—total nitrogen content in the liquid fraction of pig manure, mg kg^−1^
$X2_{1}$—mass of total nitrogen in the sludge depending on the primary sedimentation time, t
m2—mass of total nitrogen initially added with the activated sludge to the aeration tank, t

(5) $$M3=\frac{P\times Q\times 10^{-6}+m3-X3_{1}}{100}\times {\left(5.7+1.4\times \ln t_{21}\right)}^{2}$$

M3—mass of total phosphorus in the cleaned water and activated sludge after aeration, t
$t_{21}$—aeration time, d
Q—mass of the liquid fraction of pig manure, t d^−1^
P—total phosphorus content in the liquid fraction of pig manure, mg kg^−1^
m3—mass of total phosphorus initially added with the activated sludge to the aeration tank, t
$X3_{1}$—mass of total phosphorus in the sludge depending on the primary sedimentation time, t.

A multilevel model of the technology for multi-stage processing of the liquid fraction of pig manure is presented by dependencies (6):

(6) $$X1_{i}=f\left(ti,\;Q\right),\;Q\in \left(50,300\right)\;X2_{i}=f\left(ti,\;N,\;Q\right),\;Q\in \left(50,\;300\right),\;N\in \left(2000,\;6000\right)\;X3_{i}=f\left(ti,\;P,\;Q\right),\;Q\in \left(50,\;300\right),\;P\in \left(500,\;1500\right)$$

where

$X1_{i}$—mass distribution on the i-th technological operation, t
$X2_{i}$—nitrogen distribution on the i-th technological operation, t
$X3_{i}$—phosphorus distribution on the i-th technological operation, t
ti—time of the i-th operation
i= 1—technological operation of primary sedimentation
i = 2—technological operation of aeration $t_{21}$ with secondary sedimentation $t_{22}$
i = 3—technological operation of long-term storing (maturing)
$t_{21}\in \left(1;21\right)$, days
$t_{22}\in \left(1;6\right)$, hours
$t_{3}\in \left(0;6\right)$, months
Q—mass of the liquid fraction of pig manure, t day^−1^
N—total nitrogen content in the liquid fraction of pig manure, mg kg^−1^
P—total phosphorus content in the liquid fraction of pig manure, mg kg^−1^.

These dependencies helped select an optimal set of technological solutions for the multi-stage processing of pig manure to achieve the required quality and quantity of the end-product—cleaned water.

The established mathematical dependencies provide a way to estimate the adaptability of the technology to the farm resources and consumer requirements; to identify the main operation modes and the possible variation boundaries of the coefficients characterising the operation modes in the practical technology implementation.

The experimental data were analysed in Statgraphics Centurion 16.1.11. The correlation coefficient was 0.98, the determination coefficient was 96%.

##### The Fermentation of the Solid Fraction of Pig Manure

The subject of another separate experimental study was the fermentation of the solid fraction of pig manure in a laboratory drum fermenter with the solid organic fertiliser as the end-product [52,53].

The manure was separated into fractions on a pig farm by a screw separator. Before loading the solid fraction into the laboratory fermenter, the mass was measured and samples were taken to determine the moisture, total nitrogen and total phosphorus content. The fermentation lasted for 7 days. The fermenter was installed on a strain gauge balance. The measurements of the mass and the sampling took place every day. The experiment was performed in three replications. The experimental data were analysed in Statgraphics Centurion 16.1.11.

The results of the experiment were used to establish dependencies between the mass of resulting organic fertiliser, its total nitrogen and phosphorus content and the fermentation time:

(7) M=1293.98−t×17.83

where

M—the mass, kg;t—fermentation time, d.

The correlation coefficient was 0.98.

(8) P=3439.2+t×55.1

P—total phosphorus content in the resulting organic fertiliser, mg·kg^−1^.

The correlation coefficient was 0.98, the determination coefficient was 97.6%.

(9) $$N=7004.9-t\times 1649.15+t^{2}\times 905.9-t^{3}\times 157.6+t^{4}\times 8.9$$

where

N—total nitrogen content in the resulting organic fertiliser, mg·kg^−1^.

The determination coefficient was 99.8%.

#### 2.2.4. Calculation Methods for the Distribution of Produced Solid and Liquid Organic Fertilisers by Agricultural Land

The study applied the mathematical apparatus of the transportation problem of organic fertiliser transfer from supplying farms to consuming farms. The target function was the transportation cost of one ton of total nitrogen [4].

### 2.3. Methodological Approach

**Figure 4:**
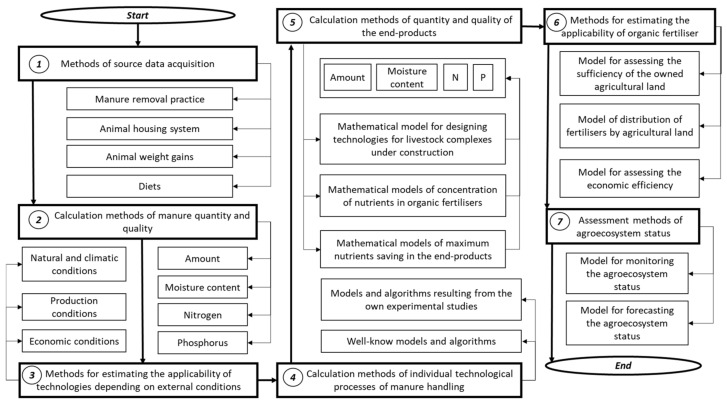
General methodological approach to designing the technologies for the end-product production.

The optimal nutrient balance in the agroecosystem means that nutrients return to the production cycle with minimal loss to the environment. Figure 4 outlines the methodological framework for assessing the manure nutrient balance and the approaches to designing the technologies for the end-product production.

Step 1 is to acquire the initial data on the pig farm. Step 2 is to calculate the quantity and quality of pig manure as a raw material with due account for the technologies in place. Step 3 is to identify the applicability of different technological solutions depending on natural and climatic conditions and the specific performance characteristics of the pig farm. Step 4 is to make a graphic representation of correlations between applicable technological solutions within the pig manure processing technology into the end-products. Step 5 is to calculate the quantity and quality of the end-products by mathematical models and to substantiate the optimal for introduction technology. Step 6 is to estimate the application options of remained organic fertiliser and to calculate the economic efficiency. The information obtained about the suggested technology can be used for a comprehensive agro-environmental and economic assessment, including the future estimation of greenhouse gas emissions and the possibility of using such a resource as clean water (step 7).

The established system of methods provides a way to assess the level of environmental compliance of pig farms and design an effective set of machines and technologies for the environmental sustainability of these farms as agroecosystems.

**Figure 5:**
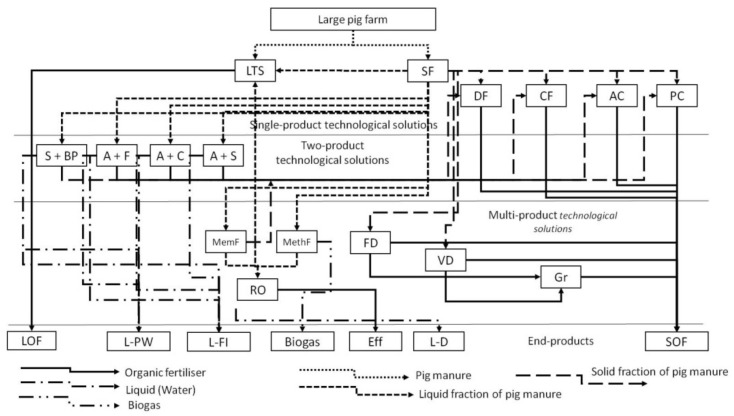
General procedure of designing the technologies to obtain the end-products from pig manure: LTS (M)—long-term storing (maturing); SF—separation into fractions; S + BP—sedimentation in tanks with stop-logs and treatment in biological ponds; A + F—aeration and flocculation; A + C—aeration and coagulation; A + S—aeration and sedimentation in batch-type settling tanks; DF—fermentation in drum fermenters; CA—fermentation in chamber fermenters; PC—passive composting; AC—active composting; MemF—membrane filtration; MethF—methane fermentation; RO—reverse osmosis; TD—thermal drying; VD—vacuum drying; Gr—granulation; LOF—liquid organic fertilizer; L-PW—liquid (water) suitable for use in the processes; L-FI—liquid (water) suitable for additional fertilizing of fodder crops; Eff—methane tank effluent; L-D—cleaned liquid (water) dischargeable into the open water bodies; SOF—solid organic fertiliser.

A comprehensive analysis of existing pig manure handling solutions with the use of the calculated coefficients made it possible to draw up a general procedure to obtain the end-products from pig manure fractions with specified nutrients content by the following methods: natural biological processing; a combination of physical actions accompanied by natural biological processes in separated pig manure fractions; a combination of physical actions and specially organised biological processes; and a combination of physical and chemical actions and organised biological processes (Figure 5).

### 2.4. Methodology Testing

The developed methodology was tested on the data from a selected full-cycle pig farm with a total pig stock of 17,800 heads and annual pig manure output of 54,750 t located in the Leningrad Region, i.e., in the Russian part of the Baltic Sea catchment area. Its emissions and diffuse inputs of nutrients were of special importance to be estimated in terms of marine environment protection [54]. A technological solution to pig manure handling in place was analysed. The promising technologies were suggested that could be introduced on the farm based on its initial resources and limiting factors.

## 3. Results

### 3.1. Selection of Technological Solutions for a Pilot Pig Farm

To analyse the substantial amount of data, we designed a computer-aided programme for assessing and selecting adaptive technologies for pig manure processing, which was approved by the State Certification Authority.

The developed methodology was applied to a full-cycle combined-type large pig farm with a total pig stock of 17,800 heads and annual pig manure output of 54,750 tons located in the Leningrad Region.

The animals were housed on a partially slatted floor without bedding except for the suckling piglets. The pig manure was removed from the livestock houses by the gravity system of batch type.

At that time, the pig farm separated manure into fractions, with the long-term storing (maturing) of the liquid fraction and passive composting of the solid fraction. The resulting solid organic fertilisers were applied to the owned agricultural land in the correct doses. The resulting liquid organic fertilisers were applied to the owned agricultural land in significantly higher doses.

The main limiting factor of the pig farm was the lack of sufficient agricultural land for the application of organic fertilisers. At least 1800 hectares of agricultural land were required to apply all the fertiliser obtained. For cost-effective fertilisation, the distance between the fields and the farm should not exceed 15 km. However, on the farm only 1280 hectares at a distance of above 15 km from the manure processing site were available.

The study objective was to apply the developed methodology to identify how to produce more concentrated fertiliser for transportation in smaller volumes over long distances.

The first step was to calculate the daily pig manure output, its moisture content and the initial content of total nitrogen and total phosphorus in the amount of pig manure transferred to processing by the values from regulatory documents. The calculation results were 150 t d^−1^ of pig manure with 93.4% moisture content, 795 kg of total nitrogen content and 247 kg of total phosphorus content.

The second step was to analyse the natural and climatic conditions, soil properties in the location of the selected pig farm and the technical possibility (land availability) to introduce the possible technological solutions for obtaining the manure-based end-products (see Figure 5). Six technologies were found most applicable on this particular farm in the Russian part of the Baltic Sea catchment area with due regard to HELCOM recommendations [54]:

Technology 1—long-term storing (maturing) (LTS (M));
Technology 2—separation into fractions (SF) + long-term storing (maturing) (LTS (M)) + passive composting (PC);
Technology 3—separation into fractions (SF) + sedimentation in tanks with stop-logs and treatment in biological ponds (S + BP) + passive composting (PC);
Technology 4—separation into fractions (SF) + aeration and flocculation (A + F) + passive composting (PC);
Technology 5—separation into fractions (SF) + aeration and coagulation (A + C) + passive composting (PC);
Technology 6—separation into fractions (SF) + aeration and sedimentation in batch-type settling tanks (A + S) + passive composting (PC).

The economic and environmental indicators were calculated for the selected technologies (Table 1).

The amounts of nitrogen and phosphorus in the end-products calculated for the above six technologies are the nutrients saved in the end-products (organic fertilisers) taking into account the losses at each technological stage—manure processing, temporary accumulation of end-products and transportation of end-products to their application place.

The area required to apply the calculated amount of fertiliser nutrients was compared with the area available on the farm.

The third step was to choose the optimal technology by the method of multi-criteria Pareto optimisation [55] (Table 2). The criteria chosen for the assessment were an economic indicator—the sum of specific capital and operational costs, and an ecological indicator—the sum of total nitrogen and total phosphorus in the end-products.

According to the ecological indicator, technology 1—long-term storing (maturing) was the most optimal for the pilot pig farm. However, the available land was not enough for its implementation. The organic fertilisers needed to be transported at disadvantageous distances, resulting in a higher economic indicator.

The developed computer-aided programme found technology 6 (separation into fractions + aeration and sedimentation in batch-type settling tanks + passive composting) the most suited for the pilot pig farm following the selected criteria and resources (insufficient area of agricultural land within economically rational transportation distance).

The programme also identified the equipment required for this technology, calculated its number, dimensions and maintenance conditions [56] (Table 3). It also specified the requirements for the dimensions of facilities (structures) (Table 4).

**Figure 6:**
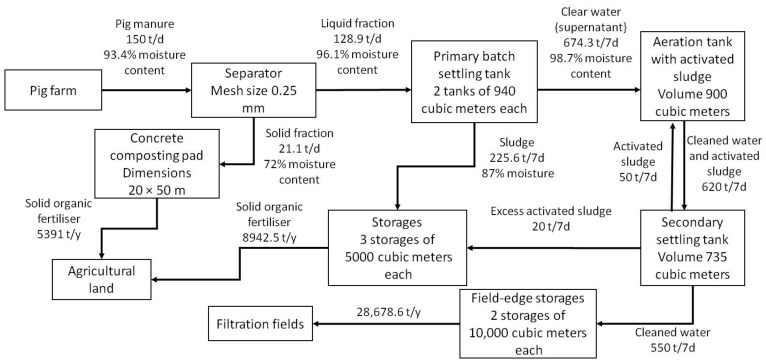
A balance diagram of the selected technology.

A balance diagram of the selected technology was created (Figure 6) to determine the quantity of the produced organic fertilisers and calculate the fertilisation doses.

### 3.2. Testing of the Developed Methodology on Pig Farms

To verify the developed methodology, the calculated data on 15 large pig farms in Russia, with seven of them being located in the Russian part of the Baltic Sea catchment area, were compared with the data from previous surveys of these farms when elaborating technological regulations for manure management.

To ensure data reliability, the initial data were acquired and analysed through a questionnaire survey of farms, examining the technological maps and technological processes as well as the protocols of laboratory analyses supplied by the accredited laboratories. The adequacy of acquired data was verified by organising field surveys of 8 out of 15 pig farms, including the sampling and sample analysis with the methods commonly used in Russia and Europe.

The obtained experimental data were statistically analysed. The mean values and standard deviations (SD) were calculated. The average experimental values for all pig farms fell in the interval of standard deviations. Therefore, the data were considered reliable (Table 5).

Pig farm 1 is located in the Kaliningrad Region; pig farms 2–7 are located in the Leningrad Region; pig farm 8 is located in the Pskov Region; pig farms 9 and 10 are located in the Republic of Buryatia; pig farms 11–13 are located in the Krasnoyarsk Territory; pig farm 14 is located in the Tomsk Region and pig farm 15 is located in the Kaluga Region.

The pig housing systems and technologies for manure removal and processing into end products were considered. The developed methodology was applied in calculations (Table 6).

From Table 6, the maximum difference between the calculated values and the actual data is 12.5% in the end-product amount, 13.9% in the end-product total nitrogen content and 9.9% in the end-product total phosphorus content.

These differences are not significant in the aggregated assessment of the large-scale intensive pig farms. Accordingly, the developed methodology can be used for an aggregated assessment of the end products based on pig manure both at the farm, district or region level.

## 4. Discussion

The intensification of pig farming is a current worldwide trend. The major challenge for intensive large-scale pig farms in Russia is to process and utilise the large amounts of manure with minimal environmental pressure (manure nutrient loss) under limited owned agricultural land and too-long transportation distances. An effective way is to choose the relevant technological manure processing solutions that would provide the balanced N and P transfer (flow) from the raw manure to the target end-products with specified characteristics (solid and liquid organic fertilisers, biogas, cleaned water, etc.) We designed a special tool (methodology) to support this choice through calculation of quantity and quality of the fresh manure and the end-products (in terms of the customer needs), the computational analysis of applicable technological solutions, the modelling of mass and nutrient distribution and the estimation of relevant costs.

The methodology is the first basic step in creating a comprehensive system of designing and effective management of pig farms based on available resources, maximum nutrient saving, limiting factors, economic and environmental considerations. This instrument is also expected to support the decision-making on the regional level on the subsidizing of farms in the transition to Best Available Techniques and the environmental permitting process. In the future, it may be applied together with the terrain estimates to design new pig farms and forecast the possible adverse environmental effects at the farm, district or region level.

A limitation of our methodology lies in some initial data acquisition for calculations—we used the literature data on soil characteristics when calculating the organic fertiliser application rates and the data provided by the pig farms on target yields. The required amount of nutrients was calculated from the nutrient removal with the yields. In the future, on-farm agrochemical soil surveys shall be provided.

The methodology was developed with reference to a large-scale pig farm in the Russian part of the Baltic Sea catchment area. The best-suited manure processing technological solution was identified to provide the most efficient environmental and economic performance.

The methodology was further tested on 15 large pig farms located in different climatic zones of Russia with their specific animal diets depending on the region, farm profile, feed base and production capacity. The calculated data based on the aggregated coefficients were compared with the verified data from previous surveys of these farms. According to the test results, the methodology can be applied to an aggregated assessment of the pig manure-based end products. However, more accurate calculations require additional examining of pig farms.

The methodology integrated several methods, calculation and modelling techniques used by researchers in other countries.

First of all, this is a digital tool based on the mass balance method, which considers the nutrient flow throughout the production chain—from feed to pig excrement and organic fertilisers [5,6,7,57]. This tool was jointly developed within the MANURE STANDARDS project of the European program “Interreg Baltic Sea Region 2014–2020”. The European national calculation tools are mostly similar to the models developed in Denmark [19]. However, they may differ in the level of detail, the algorithms used and especially the coefficients [8].

We also applied the European calculation method through nitrogen use efficiency (NUE) [1,5,7,8,18,22], with specific Russian indicators of intensive pig farming serving as initial data.

Many researchers use the modelling to predict the pig manure evolution in terms of mass, dry matter and nutrient content and related gaseous emissions. However, they either consider the flows from fresh manure to compost ready for field application as [17] or from pig excreta to manure stored before spreading, depending on different housing systems as [58].

Our methodology suggests the optimal technological solutions based on the customer farm needs: crop farms need organic fertilisers; an industrial enterprise needs biogas; the landless pig farm needs to accumulate all the nutrients in a smaller volume of organic fertiliser for lower costs of long-distance transportation, etc.

An idea of our methodology originating from the lack of agricultural land to consider other end products from pig manure with increased nutrient content and more feasible export to other farms (districts) resonates with other studies. In [59] the solid fraction of codigested manure is processed into a concentrated P fertiliser and a nutrient-poor organic soil improver; in this case, the recovered P fertiliser could be used as a secondary raw material for fertiliser production or export whereas the soil improver could be applied on arable soils in the nearby region. In [23] an advanced technical solution (reverse osmosis) is suggested to improve the pig manure management in terms of liquid fraction processing.

To improve the pig manure management systems, the researchers suggest different approaches. The authors of [33] apply Best Available Techniques Not Entailing Excessive Costs (BATNEEC) while our methodology considers all applicable technological solutions, regardless of financial investments and consequently, economic costs are calculated for these technological solutions.

To estimate the current pig manure management and suggest improvements, many researchers use the life cycle assessment (LCA) [14,15,16,60,61,62] applying different criteria, predominantly acidification potential (AP), eutrophication potential (EP) and global warming potential (GWP). This method is widely used in Europe. In our methodology, we used it only in a small block considering that all impacts are equally important between themselves. This simplification allows us to give only an aggregated estimate. In the future, a more detailed analysis of the regions is needed to obtain verified experimental numerical values.

In general, the designed methodology seems to match the European search for the most efficient use of manure nutrients through producing various end-products of manure processing.

It also could support achieving some international goals of Russia, namely, to reduce the nutrients inputs into the Baltic Sea according to the target indicators specified by the HELCOM Baltic Sea Action Plan [54,63]. On the example of intensive pig farming in the Russian part of the Baltic Sea catchment area, this study will support a better understanding of how to reach the goals and objectives of the 2021 update of the HELCOM Baltic Sea Action Plan in terms of the nutrient inputs to the water bodies [64].

## 5. Conclusions

A methodology was developed integrating methods, procedures and models to assess the environmental performance of large intensive pig farms. It was applied to a full-cycle pig farm in the Leningrad Region (Russia). Six options of pig manure processing into the target end-products were selected in terms of natural, technical and economic conditions and the limiting factors of the farm. The best suitable technology was identified, with the economic indicator, i.e., the sum of specific capital and operational costs, being USD 55.5 per ton of pig manure and the ecological indicator being 278.94 t of total nitrogen and phosphorus in the end-products per year.

The methodology was further tested by comparing the calculated data on 15 large pig farms in Russia with the verified data from previous surveys of these farms when elaborating technological regulations for manure management. The maximum difference between the calculated values and the actual data was 12.5% in the end-product amount, 13.9% in the end-product total nitrogen content and 9.9% in the end-product total phosphorus content. These differences are not significant in the aggregated assessment of large-scale intensive pig farms.

The study results are expected to become the starting methodological point for the creation of advanced waste management systems in intensive pig farming through a comprehensive approach to producing different end-products by the most effective manure processing technology and with the least manure nutrient loss to the environment at the farm, district and regional level.

## Figures and Tables

**Table 1:** Estimated economic and ecological indicators of obtaining the end-products on the pilot pig farm.

Indicators	Technology 1	Technology 2	Technology 3	Technology 4	Technology 5	Technology 6
Electrical energy costs, USD t^−1^ y^−1^	0.1	0.1	0.004	1.3	1.6	2.1
Fuel costs, USD t^−1^ y^−1^	12.8	11.5	8.6	7.7	8.6	6
Labour costs, USD t^−1^ y^−1^	6.4	3.8	4.3	4.8	3.4	3
Specific capital costs, USD t^−1^ y^−1^	49.4	43.9	49.1	38.2	43.8	41
Specific operational costs, USD t^−1^ y^−1^	38.5	17.3	19.2	40.9	18.8	14.4
Amount of total nitrogen in the end-products, t y^−1^	265.71	208.34	120.78	196.27	175.13	214.38
Amount of total phosphorus in the end-products, t y^−1^	70.71	62.26	57.64	60.72	56.88	64.56
Liquid organic fertiliser produced, t	44,331.47	43,073.75	–	–	–	–
Solid organic fertiliser produced, t	–	4539.51	8231.55	10,265.01	9744.72	14,333.5
Cleaned water produced, t	–	–	34,837.67	22,115.75	28,425.56	28,678.57

**Table 2:** The choice of the optimal technology with due account for the technical and land resources of the pig farm.

Indicator	Unit	Direction of Extremum	Technology 1	Technology 2	Technology 3	Technology 4	Technology 5	Technology 6
Economic indicator	USD t^−1^ y^−1^	Min	87.9	61.2	68.4	79.1	62.6	55.5
Ecological indicator	t y^−1^	Max	336.42	270.6	178.42	256.99	232.01	278.94

**Table 3:** Required equipment for the selected technology of pig manure processing.

Equipment	Specifications	Amount Required
Covered batch-type settling tank for primary sedimentation	Dimensions—6.5 × 50 × 3 m	2
Front-end loader	The basket volume at least 3 m^3^	2
Mixer	Power consumption—5.5 kW h^−1^	1
Pump	Throughput—50 t h^−1^ Power consumption—5.5 kW h^−1^	4
Secondary sedimentation tank	Dimensions—5.1 × 50 × 3 m	1
Separator	Throughput—15 t h^−1^ Power consumption—4 kW h^−1^	1
Tractor	Draw-bar capacity—1.4 to 2.0 tf (12.6–27.0 kN) (ISO drawbar category 2)	2
Trailer	Capacity, 10 t	2
Trailer-spreader	Capacity, 10 t	2
Two-section aeration tank	Dimensions of one section—3 × 50 × 3 m Two air blowers	1

**Table 4:** Required facilities (structures) for the selected technology.

Facility (Structure)	Specifications	Amount Required
Concrete pad for passive composting of the solid fraction of pig manure	Dimensions—110 × 125 m Technological passages between the compost piles are 5 m wide Technological passages between the pad edge and a pile are 10 m wide	1
Concrete storage for long-term maturing of the sludge and excess activated sludge	Each storage dimensions of 33 × 50 × 3 m One of the storage walls should be inclined for the access of vehicles to unload the ready solid organic fertiliser	3
Concrete storages for accumulation of cleaned water from October to April	With the capacity of 10,000 m^3^ each will be used	2
Filtration fields	Arranged with the Code 32.13330.2018 “Sewerage. Pipelines and wastewater treatment plants”	42.5 ha

**Table 5:** Statistical analysis of the data from the laboratory protocols on the total nitrogen and phosphorus content in the pig manure-based end-products.

Pig Farm	Type of End-Product	Nitrogen Amount in the End-Products, mg/kg			Phosphorus Amount in the End-Products, mg/kg		
		Average Experimental Data $\bar{x}$	$\bar{x}+\sigma$	$\bar{x}-\sigma$	Average Experimental Data $\bar{x}$	$\bar{x}+\sigma$	$\bar{x}-\sigma$
Pig farm 1	Solid organic fertiliser	2800	2814	2786	–	–	–
	Liquid organic fertiliser	2100	2117.5	2082.5	–	–	–
Pig farm 2	Solid organic fertiliser	3200	3227.3	3172.7	900	912.1	900
	Liquid organic fertiliser	2200	2209.8	2190.2	700	706.4	693.6
Pig farm 3	Solid organic fertiliser	2900	2911.8	2888.2	850	855.4	844.6
	Liquid organic fertiliser	2157	2164.2	2149.8	730	734.7	725.3
	Aqua ammonia	56,000	56,057.5	55,942.5	0	0	0
	Effluent for additional fertilisation of grass	860	868.1	851.9	290	295.3	284.7
Pig farm 4	Liquid organic fertiliser	2500	2521.8	2478.2	710	715.2	704.8
Pig farm 5	Solid organic fertiliser	3080	3093.4	3066.6	870	877.6	862.4
	Liquid organic fertilisers	2250	2259.9	2240.1	650	656.5	643.5
Pig farm 6	Liquid organic fertiliser	2290	2314.6	2275.4	–	–	–
Pig farm 7	Liquid organic fertiliser	2460	2475.7	2444.3	–	–	–
Pig farm 8	Liquid organic fertiliser	1970	1989.3	1950.7	640	648.5	631.5
Pig farm 9	Solid organic fertiliser	5400	5426.9	5373.1	–	–	–
	Effluent for additional fertilisation of grass	1500	1507.1	1492.9	–	–	–
Pig farm 10	Solid organic fertiliser	4600	4621.7	4578.3	–	–	–
	Liquid organic fertiliser	1900	1909.7	1890.3	–	–	–
Pig farm 11	Liquid organic fertiliser	2060	2066.4	2053.6	610	612.1	607.9
Pig farm 12	Liquid organic fertiliser	2240	2251.2	2228.8	635	639.5	630.5
Pig farm 13	Liquid organic fertiliser	2800	2816.2	2783.8	670	676.9	663.1
Pig farm 14	Solid organic fertiliser	2700	2714.8	2685.2	–	–	–
	Liquid organic fertiliser	2350	2359.3	2340.7	–	–	–
Pig farm 15	Liquid organic fertiliser	2300	2315.6	2284.4	740	747.2	732.8

**Table 6:** Estimated and actual indicators of final products based on pig manure.

Pig Farm	Type of End-Product	Amount of End-Products			Nitrogen Amount in the End-Products			Phosphorus Amount in the End-Products		
		Actual, t/y	Calculated, t/y	Difference, %	Actual, t/y	Calculated, t/y	Difference, %	Actual, t/y	Calculated, t/y	Difference, %
Pig farm 1	Solid and liquid organic fertilisers	19,174	18,019.9	6	36.5	37.7	3.3	–	14.8	–
Pig farm 2	Solid and liquid organic fertilisers	19,381.5	19,214.5	0.9	47.1	43.2	8.3	16.7	16.1	3.6
Pig farm 3	Solid and liquid organic fertilisers, aqua ammonia, effluent for additional fertilisation of grass	18,2930	16,0152.3	12.5	339.1	291.9	13.9	175.7	158.3	9.9
Pig farm 4	Liquid organic fertilisers	44,906.4	40,745.5	9.3	130.2	126.6	2.8	31.4	33.3	6.1
Pig farm 5	Solid and liquid organic fertilisers	40,405.5	41,919.8	3.7	128	112.4	12.2	52.6	53.5	1.7
Pig farm 6	Liquid organic fertilisers	12,702	11,872.5	6.5	27.4	23.7	13.4	–	7	–
Pig farm 7	Liquid organic fertilisers	98,700	88,850.1	10	236.9	222.7	6	–	65.9	–
Pig farm 8	Liquid organic fertilisers	113,127.2	114,483.6	1.2	214.9	231.7	7.8	67.9	72.4	6.6
Pig farm 9	Solid organic fertilisers and effluent for additional fertilisation of grass	68,440.4	62,356.8	8.9	128	120.3	6	–	41.8	–
Pig farm 10	Solid and liquid organic fertilisers	194,780.6	184,653.1	5.2	428.4	379.7	11.4	–	105.9	–
Pig farm 11	Liquid organic fertilisers	52,438.5	51,779.7	1.3	99.6	96.6	3	31.5	33.7	7
Pig farm 12	Liquid organic fertilisers	49,685.7	48,447.8	2.5	124.2	117.5	5.4	29.8	28.6	4
Pig farm 13	Liquid organic fertilisers	170,458.6	150,144.6	11.9	473.5	430.1	9.2	85.2	78.1	8.3
Pig farm 14	Solid and liquid organic fertilisers	1,025,935.2	926,502.1	9.7	1425.5	1293.9	9.2	–	441.3	–
Pig farm 15	Liquid organic fertilisers	18,700	18,365.6	1.8	43	38.5	10.5	13.1	12.4	5.3

## Data Availability

The data presented in this study are available on request from the corresponding author. The data reflect the specific conditions of agricultural enterprises that are covered by the privacy policy.
